# New Biodegradable Poly(l-lactide)-Block-Poly(propylene adipate) Copolymer Microparticles for Long-Acting Injectables of Naltrexone Drug

**DOI:** 10.3390/polym12040852

**Published:** 2020-04-07

**Authors:** Stavroula Nanaki, Athina Viziridou, Alexandra Zamboulis, Margaritis Kostoglou, Georgios Z. Papageorgiou, Dimitrios N. Bikiaris

**Affiliations:** 1 Laboratory of Polymer Chemistry and Technology, Department of Chemistry, Aristotle University of Thessaloniki, GR-541 24 Thessaloniki, Greece; sgnanaki@chem.auth.gr (S.N.); azampouli@chem.auth.gr (A.Z.); 2 Department of Food Science and Technology, International Hellenic University, GR-57400 Thessaloniki, Greece; athina_viziridou@hotmail.com; 3 Laboratory of General and Inorganic Chemical Technology, Department of Chemistry, Aristotle University of Thessaloniki, GR-541 24 Thessaloniki, Greece; kostoglu@chem.auth.gr; 4 Chemistry Department, University of Ioannina, P.O. Box 1186, 45110 Ioannina, Greece; gzpap@uoi.gr

**Keywords:** poly(l-lactide), poly(propylene adipate), amphiphilic block copolymers, naltrexone, drug encapsulation, microspheres, long-acting injectables, controlled release

## Abstract

In the present study, novel block copolymers of poly(l-lactide)-block-poly(propylene adipate) (PLLA-*b*-PPAd) were synthesized in two ratios, 90/10 and 75/25 *w*/*w* and were further investigated as long-acting injectable (LAI) polymeric matrices in naltrexone base microparticle formulations. The synthesized polymers were characterized by ^1^H-NMR, ^13^C-NMR, FTIR, XRD, TGA and DSC. NMR and FTIR spectroscopies confirmed the successful synthesis of copolymers while DSC showed that these are block copolymers with well-defined and separated blocks. Microparticles were prepared by single emulsification method and were further characterized. Nanoparticles in the range of 0.4–4.5 μm were prepared as indicated by SEM, with copolymers giving the lowest particle size. By XRD and DSC it was found that naltrexone was present in the amorphous state in its microparticles. Dissolution study showed a drug release extending over seven days, indicating that these novel PLLA-*b*-PPAd copolymers could be promising matrices for naltrexone’s LAI formulations. It was evidenced that drug release depended on the copolymer composition. Model release studies showed that drug release is controlled by diffusion.

## 1. Introduction

Aliphatic polyesters have proven to be a very promising class of biomaterials. Most importantly, these polyesters are regarded as readily biodegradable, due to the hydrolysable character of the ester bond and its accessibility (unlike aromatic polyesters). Additionally, aliphatic polyesters are generally biocompatible, due to the benign initial reagents (acids, hydroxy acids or diols), and to their biodegradation products which are water-soluble oligomers. Both initial monomers and degradation products are easily eliminated through urine cell activity [1]. As a result of their biodegradability and biocompatibility, another major advantage of aliphatic polyesters when it comes to drug delivery, is that there is no need for further surgical removal after drug exhaustion. Furthermore, aliphatic polyesters can easily be modified in order to tune the release of the encapsulated drug and achieve a desirable drug delivery profile [2]. Typical aliphatic polyesters that have already been studied as drug carriers are ε-polycaprolactone (PCL), poly(lactic acid) (PLA), poly(glycolic acid) (PGA), their copolymer poly(lactide-co-glycolide) (PLGA), which are typically prepared by ring-opening polymerizations, and polyesters resulting from the polycondensation of an aliphatic diol and an aliphatic dicarboxylic acid such as poly(ethylene succinate), poly(ethylene adipate), poly(propylene adipate) (PPAd). Among them, PLA is by far the most extensively studied. However, the use of PLA suffers from some limitations due to its high crystallinity and low hydrolysis rate. Copolymerization is an easy method to modulate the properties of a polymer, by varying the rigidity/flexibility of polymer chains, the hydrophilic/hydrophobic balance and the amorphous/crystalline ratio [3]. Copolymerization of PLA with more hydrophilic and amorphous polymers has been carried out to modify the required biomaterial properties for specific applications [4].

Naltrexone base (NTX) is a specific opioid antagonist, used in the treatment of both drug addiction and alcohol dependence. It is marketed in tablet form for oral administration but has some pharmacotherapeutic limitations (for example, a low oral bioavailability, as 98% of the drug is metabolized in the liver) and gastrointestinal side effects (nausea, abdominal pain, and vomiting). Treatment by oral administration of NTX requires a daily administration and its efficacy can be compromised due to the patient’s non-compliance. Indeed, 37% of patients discontinue the daily oral use of NTX by 12 weeks and more than 80% discontinue its use by six months [5]. Currently there are two major types of sustained-release formulations for NTX delivery: (a) injectable depot formulations for long-acting release (Naltrel^®^, Vivitrol^®^, and Depotrex^®^), and (b) surgically implantable pellets (Prodetoxone®, Wedgewood®, and O’Neil®) [6]. Regarding NTX implants, an early period (up to 12 months post-implant) of inflammation, foreign body reaction, and fibrosis has been reported, while the high cost of the implant limits its use [7]. Thus, novel drug delivery systems, such as microspheres and nanoparticles for long-acting injectables (LAI), that are able to be administered by parenteral route, are expected to improve NTX therapy. Indeed, such systems would allow the elimination of the first-pass metabolism, while providing a prolonged release. As a result, of the sustained prolonged release in LAI, a lower dosage could be achieved, thus reducing the toxic secondary effects. Finally, a single parenteral administration could tackle patient non-compliance issues.

Microparticle-formulations of NTX have already been reported, several of which involve PLA copolymers. For example, Akala et al. prepared and studied NTX-loaded poly(lactide-*co*-glycolide) (PLGA) microspheres [8]. The influence of the molar composition and the average molecular weight of the copolymers on the formation of nanospheres and the sustained release of NTX was studied. Depending on the formulation, release ranged from 30 to 150 days. Salehi et al. developed NTX-loaded micelles based on poly(N-isopropylacrylamide)-b-poly(l-lactide) copolymers [9]. It was shown that the increase of PLA resulted in a higher drug loading of the micelles, but also to a slower release (5 vs. 18% within the first 24 h). Pagar et al. introduced 5 mol% of a cyclic glycolic acid/leucine monomer (depsipeptide) to lactide polymerization to obtain a lactide-depsipeptide copolymer [10]. Microspheres loaded with NTX were formed employing a single emulsion solvent evaporation technique. NTX release studies showed an 82% release within 30 days. Nerantzaki et al. synthesized a series of castor oil, poly(ε-caprolactone) and poly(ethylene glycol) (PEG) block copolymers [11]. These copolymers were used as polymeric carriers for the fabrication of NTX-loaded microspheres by the single emulsion solvent evaporation technique, resulting in sustained release systems with drug release controlled by diffusion processes. Finally, similar PLGA-PEG-PLGA tri-block copolymers were also used as a delivery matrix, for the controlled release of naltrexone hydrochloride [12]. In vivo experiments showed that naltrexone half-life was successfully increased.

In the present study, novel poly(l-lactide)-block-poly(propylene adipate) copolymers, with two different mass compositions (90/10 and 75/25 *w*/*w*) were synthesized for the first time, as far as our knowledge goes. PLLA-*b*-PPAd copolymers were used as polymeric matrices for the nanoencapsulation of naltrexone drug, by the single emulsification method. The sustained release of NTX was studied, exhibiting a diffusion-controlled release mechanism. Finally, the experimental release studies were complemented by a theoretical modeling.

## 2. Materials and Methods

### 2.1. Materials

Adipic acid (AdA) (purity: >99.5%), poly(phosphoric acid) (PPA) and 1,3-propanediol (1,3-PD) (purity: >99.6%) were purchased from Fluka (Steinheim, Germany). Titanium butoxide (purity: >97.0%), Ti(OBu)_4_) and tin(II) 2-ethylhexanoate (Sn(Oct)_2_) were used as catalysts, l-Lactide (LA), (3*S*)-*cis*-3,6-Dimethyl-1,4-dioxane-2,5-dione (98%) and poly(vinyl alcohol) (PVA), *M*_w_ 31,000–50,000 Da (87%–89% hydrolyzed) were purchased from Sigma Aldrich Chemical Co (Steinheim, Germany). *Rhizopus oryzae* and *Pseudomonas cepacia* were purchased from BioChemika (Steinheim, Germany). Naltrexone base (99.9%) was kindly donated by Pharmathen S.A. (Athens, Greece). All other reagents and solvents used for the analytical methods were of analytical grade.

### 2.2. Synthesis of Aliphatic Polyesters

#### 2.2.1. Synthesis of Poly(Propylene Adipate) (PPAd) Aliphatic Polyester

The classic two-stage melt polycondensation method was used for the synthesis of PPAd aliphatic polyesters [13,14]. In the first stage (esterification), adipic acid (AdA) and 1,3-propanediol (1,3-PD), in a 1/1.2 molar ratio were charged into the reaction tube in the presence of 400 ppm of Ti(OBu)_4_. The reaction mixture was heated at 180 °C, in a nitrogen atmosphere, under continuous stirring. The reaction lasted until the theoretical amount of H_2_O produced during the reaction was collected. In the second stage (polycondensation), poly(phosphoric acid) PPA was added (5 × 10^−4^ mol of PPA/mol AdA) and vacuum (5.0 Pa) was slowly applied to reduce the creation of foam and the risk of poly(propylene adipate) oligomer sublimation. Polycondensation procedure was carried out at 240 °C for 2 h.

#### 2.2.2. Synthesis of Poly(l-Lactide)-*b*-Poly(Propylene Adipate) Copolymers

PLLA-*b*-PPAd copolymers, in 90/10 and 75/25 mass ratios, were synthesized by ring-opening polymerization of lactide. Proper amounts of LA and PPAd polyester were inserted into the reaction tube and a toluene solution of Sn(Oct)_2_ was added (400 ppm). Ring-opening polymerization took place at 220 °C, under nitrogen flow, for 60 min, while the stirring rate was kept stable at 500 rpm. At the end of the polymerization, unreacted LA monomer was removed by sublimation by slowly applying vacuum (≈5 Pa) over a period of 15 min, to avoid excessive foaming. The synthesized polyesters were further purified by dissolution in chloroform and precipitation with cold methanol.

### 2.3. Polymer Characterization

#### 2.3.1. Intrinsic Viscosity

Intrinsic viscosity, [*η*], measurements were performed with an Ubbelohde viscometer (Schott Gerate GMBH, Hofheim, Germany) at 25 °C. All polyesters were dissolved in chloroform (1% *w*/*v*) at room temperature and filtered through a 0.2 mm Teflon filter. The intrinsic viscosity was calculated using Equation (1) [15]:

(1) [η]=[2{tt0−ln(tt0)−1}]12c

where *c* = concentration of the solution, *t* = flow time of the polymer solution and *t*_0_ = flow time of pure solvent (CHCl_3_).

#### 2.3.2. Gel Permeation Chromatography (GPC)

Molecular weight determinations were performed by gel permeation chromatography (GPC) using a high temperature GPC system, Waters model 600, equipped with Shimadzu RID-10A (Milford, MA, USA) refractive index detector and columns Water Styragels (Milford, MA, USA) in the order HR5, HR4, HR3, HR2, HR1, 10 mg/700 μL sample concentration, and a 200 µL injection volume. Tetrahydrofuran (THF) was used as the eluent (1 mL/min) and the measurements were performed at 35 °C. Calibration was performed using 9 polystyrene standards with 1000–300,000 molecular weight distribution.

#### 2.3.3. Nuclear Magnetic Resonance (^1^H-NMR, ^13^C-NMR)

NMR spectra were recorded in deuterated chloroform (CDCl_3_), on an Agilent 500 spectrometer (Agilent Technologies, Santa Clara, CA, USA), at room temperature. Spectra were internally referenced with tetramethylsilane (TMS) and calibrated using the residual solvent peak. 

#### 2.3.4. X-ray Powder Diffraction (XRD)

X-ray powder diffraction (XRD) patterns of neat PLLA, PPAd, and their copolymers were recorded using a MiniFlex II diffractometer (Rigaku, model MiniFlex 600, Chalgrove, Oxford, UK), with Bragg–Brentano geometry (θ, 2θ) and a Ni-filtered Cu K radiation (λ = 0.154 nm). The samples were scanned from 5° to 55° 2θ.

#### 2.3.5. Fourier Transform-Infrared Spectroscopy (FTIR)

FTIR spectra were obtained using a Perkin Elmer FTIR spectrometer, model Spectrum 1000 (Perkin Elmer, Waltham, MA, USA). In order to collect the spectra, a small amount of polymer was grinded with KBr (1 wt%) and compressed to form tablets. The IR spectra of these tablets, in absorbance mode, were obtained in the spectral region of 450–4000 cm^−1^ using a resolution of 4 cm^−1^ and 24 co-added scans.

#### 2.3.6. Differential scanning calorimetry (DSC)

Differential scanning calorimetry (DSC) was performed using model DSC Q2000 (TA Instruments, Eschborn, Germany). Samples of 5 ± 0.1 mg were sealed in aluminum caps and heated 50 °C above their melting point, at a 20 °C/min heating rate, under nitrogen atmosphere. The samples were held at that temperature for 5 min in order to erase any thermal history and then cooled to −85 °C (cooling rate 250 °C/min) in order to prevent crystallization. The samples were heated again using the same conditions. *T*_g_, *T*_m_, and heat of fusion were recorded from the second scan.

#### 2.3.7. Thermogravimetric Analysis (TGA)

The thermogravimetric analysis was performed using the Setaram Setsys TG-DTA 16/18 instrument (Setaram instrumentation, Lyon, France). Samples of neat PPAd, PLLA and PLLA/PPAd copolymers, weighed 6.0 ± 0.2 mg, were placed in aluminum crucibles. A blank aluminum crucible was used as a reference. The samples were heated from ambient temperature to 550 °C, with a constant flow rate of 50 mL/min nitrogen at rates of 10 °C/min.

#### 2.3.8. Enzymatic Hydrolysis Study

Films of neat PLLA, PPAd and PLA/PPAd copolymers were prepared (5 × 5 cm^2^ and 5 mm thickness) with a PW 30 Otto Weber hydraulic press (Paul-Otto Weber GmbH, Remshalden, Germany) connected to a temperature controller (Omron E5AX). The samples were incubated at 37 ± 1 °C for 20 days in Petri dishes containing phosphate buffered saline (PBS) (pH = 7.4) with *Rhizopus oryzae* and *Pseudomonas Cepacia* lipases at 0.09 and 0.01 mg/mL concentrations respectively. After specific period intervals, films were removed from the Petri dishes, washed with distilled water and weighed till constant weight. The degree of biodegradation was estimated by mass loss of pre-weighed samples.

Weight loss percentage of the polyester films was obtained according to the following equation:

(2) $$Weight\;loss\;\left(\%\right)=\frac{\left(W_{0}-W_{r}\right)}{W_{0}}\times 100\%$$

where *W*_0_ is the weight of the specimens before degradation, *W_r_* the weight of the specimens after degradation and drying.

#### 2.3.9. Scanning Electron Microscopy (SEM)

SEM (JSM-6390LV, JEOL Company, Peabody, MA, USA) was used to evaluate the morphology of the prepared films prior and after enzymatic hydrolysis. Carbon coating was used to cover the obtained films (improvement in the conductivity of the electron beam), while the accelerating voltage, the probe current, and the counting time were set at 20 kV, 45 nA, and 60 s, respectively.

### 2.4. Microparticles Formulation

#### 2.4.1. Preparation of PLLA-*b*-PPAd Microparticles

An oil-in-water (O/W) emulsification technique was used for NTX microencapsulation in polyester matrices. Briefly, 150 mg of polymer and 50 mg of NTX were dissolved in 10 mL of dichloromethane (DCM). The resultant solution was added dropwise in 50 mL of a 1% *w*/*v* PVA aqueous solution. Homogenization was performed with a rotor stator stirrer (Ultra Turax IKA T18 basic, Staufen, Germany) for 2 min at 24,000 rpm. The homogenous dispersion was then transferred to 100 mL of deionized water, homogenized for 1 min using a rotor stirrer and left overnight under magnetic stirring till total evaporation of DCM. The formed microspheres were collected via centrifugation at 9000 rpm for 20 min (Thermo Electron Corporation, Model Heraeus Pico 17 Centrifuge), and washed three times with deionized water in order to remove the remaining quantities of PVA. Microparticles were isolated by freeze drying.

#### 2.4.2. Characterization of Microparticles

##### 2.4.2.1. In Vitro Release Profile

Dissolution studies were conducted using United States Pharmacopeia I basket method (50 rpm, 37 °C, and 500 mL dissolution medium). NTX-loaded microspheres suspensions corresponding to 50 mg of drug were placed in a dialysis cellulose membrane bag (D9402-100FT; Sigma-Aldrich, Steinheim, Germany) with molecular weight cut-off 12,000–14,000, placed into the baskets, and transferred to 500 mL phosphate buffer (pH 7.4) containing 0.5 mL Tween 20. Samples (2 mL) were withdrawn at predetermined intervals and filtered through 0.45 μm filters. The samples were analyzed by HPLC as described beneath (Section 2.4.2.2). The percentage yield, drug loading, and drug entrapment efficiency of microspheres were calculated according to the following equations:

(3) $$\mathrm{Yield}\;\mathrm{of}\;\mathrm{microparticles}\;\left(\%\right)=\frac{\left(\mathrm{weight}\;\mathrm{of}\;\mathrm{microparticles}\right)}{\left(\mathrm{weight}\;\mathrm{of}\;\mathrm{polymer}\;\mathrm{and}\;\mathrm{drug}\;\mathrm{fed}\;\mathrm{initially}\right)}\times 100$$

(4) $$\mathrm{Drug}\;\mathrm{loading}\;\left(\%\right)=\frac{\left(\mathrm{weight}\;\mathrm{of}\;\mathrm{drug}\;\mathrm{in}\;\mathrm{microparticles}\right)}{\left(\mathrm{weight}\;\mathrm{of}\;\mathrm{microparticles}\right)}\times 100$$

(5) $$\mathrm{Entrapment}\;\mathrm{efficiency}\;\left(\%\right)=\frac{\left(\mathrm{weight}\;\mathrm{of}\;\mathrm{drug}\;\mathrm{in}\;\mathrm{microparticles}\right)}{\left(\mathrm{weight}\;\mathrm{of}\;\mathrm{initially}\;\mathrm{used}\;\mathrm{drug}\right)}\times 100$$

##### 2.4.2.2. HPLC Analysis

Quantitative analysis was performed using a Shimadzu high pressure liquid chromatography (HPLC) prominence system consisting of a degasser (Model DGU-20A5, Tokyo, Japan), a liquid chromatograph, an auto sampler, a diode array detector and a thermostatic oven. A modified, previously validated, method was used for the analysis [9]. The chromatograms were obtained and processed with the LC solutions software v. 1.21 SP1. In detail, an Athena CNW Technology C18, (250 × 4.6 mm and 5 μm internal diameter) at column temperature of 40 °C chromatographic workstation (CSW) was used for regression analysis and data acquisition. Flow rate was 1 mL/min and the injection volume were 20 μL. Mobile phase was phosphate buffer (10 mM, pH was adjusted to 3.5 using H_3_PO_4_)/ACN 80/20 *v*/*v.* Each sample was measured in triplicate.

## 3. Results and Discussion

### 3.1. Synthesis and Characterization of Block Copolymers

**Figure 1:**
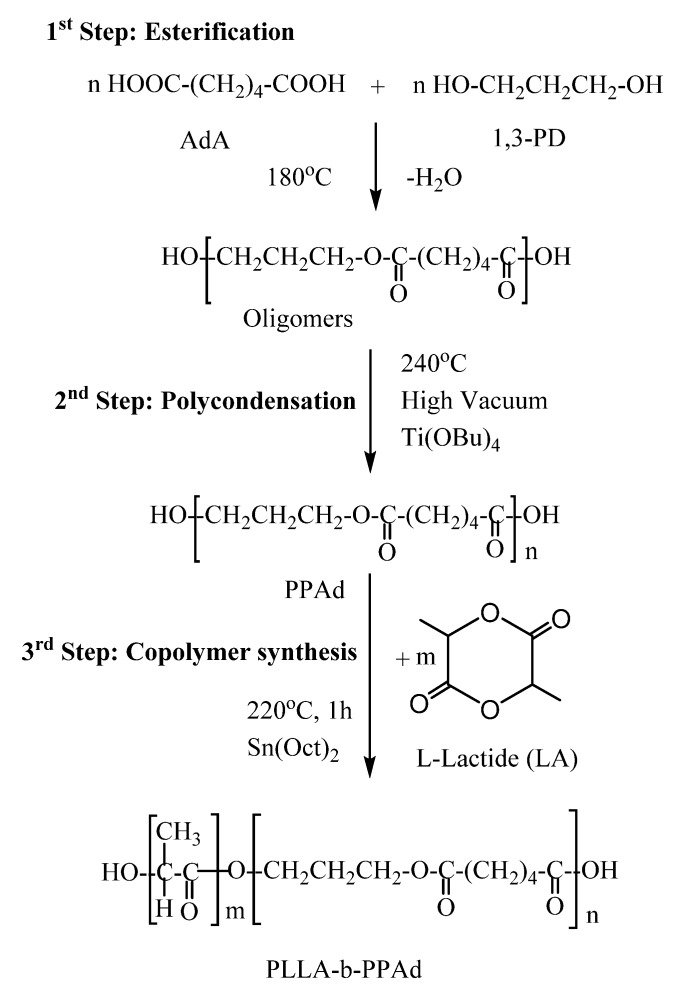
Synthesis of poly(l-lactide)-*b*-poly(propylene adipate) copolymers.

PLLA-*b*-PPAd copolymers were synthesized by a combination of melt polycondensation and ring-opening polymerization, as described in Figure 1. Initially, poly(propylene adipate) (PPAd) homopolymer was synthesized by a two-stage melt polycondensation method [16,17]. Briefly, during esterification (1st stage), PPAd oligomers were synthesized and water was removed as a byproduct. During polycondensation (2nd stage), the temperature was increased to 240 °C and reduced pressure was applied in order to build up the molecular weight of the polyester. Water and 1,3-propanediol byproducts were removed by distillation in a graduated cylinder. 1,3-propanediol was added in small excess ensuring that most of the prepared macromolecules acquired hydroxyl end groups. It is necessary to have hydroxyl-terminated macromolecules, as these will act as initiators for the ring-opening polymerization of lactide and carboxylic acid groups cannot initiate this polymerization.

The copolymerization between PLLA and PPAd was carried out at 220 °C in the presence of stannous octoate (Sn(Oct)_2_), by ring-opening polymerization of LA, using the prepared PPAd homopolymer as a macroinitiator. After 1 h at atmospheric pressure, vacuum was applied (≈5 Pa) for 15 min to remove any unreacted LA. PLLA-*b*-PPAd copolymers, containing 10 and 25 wt% of PPAd were thus synthesized (Figure 1).

**Figure 2:**
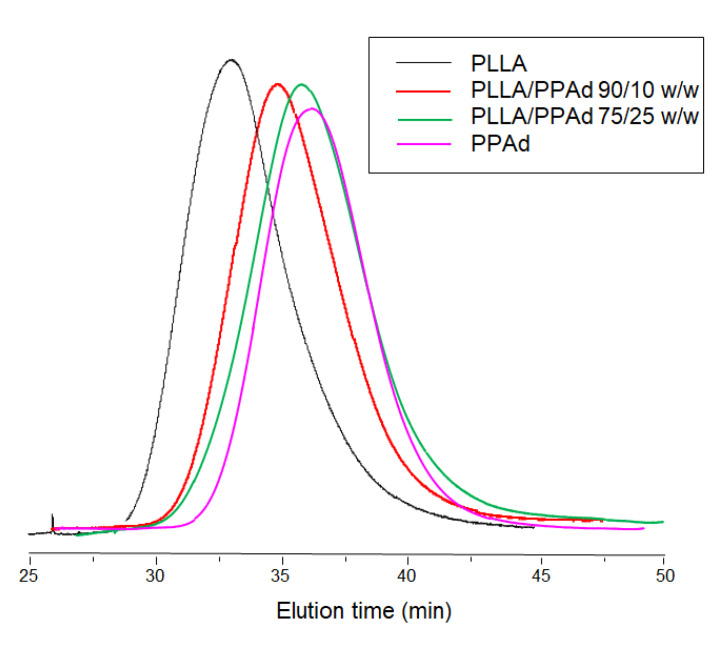
Gel permeation chromatography of poly(l-lactide) (PLLA), poly(propylene adipate) (PPAd) and PLLA-*b*-PPAd copolymers.

Characterization of the synthesized PPAd polyester by GPC measurements showed an average number molecular weight of 35,000 g/mol and an average weight molecular weight of 50,700 g/mol. The corresponding values for PLLA were 86,000 g/mol and 133,900 g/mol, respectively, while for the copolymers the recorded molecular weights ranged between 47,700–53,700 g/mol, as detailed in Table 1. Intrinsic viscosities of PLLA and PPAd were found to be 0.99 and 0.38 dL/g respectively while the values for the copolymers ranged between 0.65–0.77 dL/g. It was observed that as the percentage of PPAd increased, the intrinsic viscosity decreased. In other words, the addition of PPAd led to a decrease in the molecular weight of the formed copolymers. This was expected since the LA amount for the preparation of copolymers was reduced and thus shorter chains were obtained. All samples exhibited unimodal distribution in GPC, i.e., only a single broad peak appeared (Figure 2), with no oligomers being detected. In addition, the low polydispersity index (PDI) values indicate that no transesterification and/or backbiting reactions occurred during the copolymerization [18].

**Figure 3:**
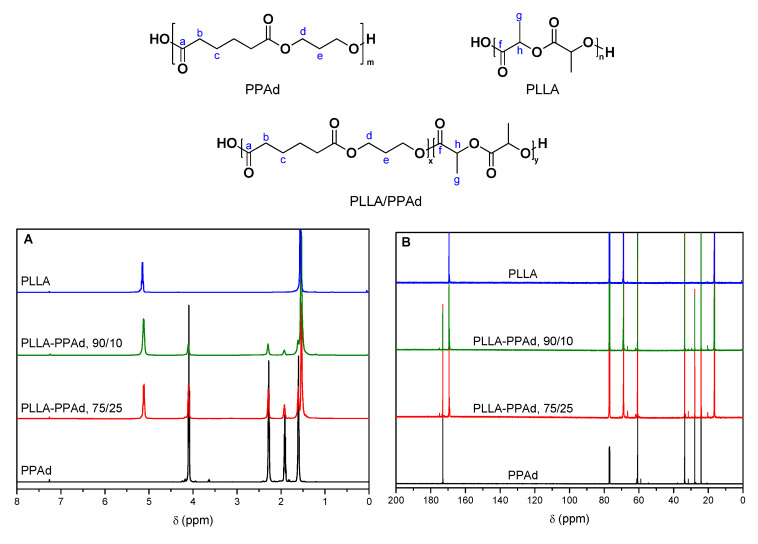
(**A**) ^1^H-NMR and (**B**) ^13^C-NMR spectra of PLLA, PLLA-*b*-PPAd copolymers and PPAd.

In order to evaluate the successful synthesis of the copolymers, NMR spectra were recorded. The ^1^H-NMR spectra of PLLA, PPAd, and PLLA-*b*-PPAd copolymers are shown in Figure 3A. The ^1^H-NMR spectrum of PPAd, exhibits a triple peak at 4.15 ppm corresponding to methylene group *d*, a quintuple peak at 1.97 ppm corresponding to methylene group *e*, a single peak at 2.33 ppm owing to methylene group *b* and a multiple peak at 1.66 ppm attributed to methylene group *c*. These peaks have also been documented in previous studies [16,17]. Typical resonance signals were obtained for PLLA, i.e., a quadruple peak at 5.17 ppm attributed to methine group *h*, and a double peak at 1.6 ppm corresponding to methyl group *g*. These peaks are in accordance with the literature [19,20].

^1^H-NMR spectra of copolymers were similar for all the copolymers synthesized, and exhibited the characteristic resonance signals of both PLLA and PPAd: methylene protons *b* and *c* at 2.29 and 1.62 ppm respectively, methylene protons *d* and *e* at 4.11 and 1.93 ppm respectively (PPAd segment) and methine and methyl protons *h* and *g* at 5.13 and 1.54 ppm respectively (PLLA segment). In the spectra of PPAd, the peak at 3.65 ppm is attributed to the protons *d′* which are adjacent to the hydroxyl end-group of PPAd chains. During the polymerization, PPAd chains initiate the ring-opening polymerization of lactide via the OH-terminated end. The disappearance of this peak in the spectra of the PLLA-PPAd copolymers indicates the successful polymerization and the formation of block copolymers. The molar composition of the copolymers was calculated by ^1^H-NMR spectra comparing the integrations of the CH(*h*) group of the PLA unit and the CH_2_(*e*) group of PPAd. For both copolymers, a better incorporation of PPAd units in the copolymers was observed (15 and 31 wt% instead of 10 and 25 wt%). This could be attributed to some lactide sublimation, due to the high temperature of polymerization. All results are presented in Table 1.

In the ^13^C spectrum (Figure 3B), the carbonyl bond of the ester groups are observed at 169.5 ppm (C=O(*f*), PLLA) and 173.1 ppm (C=O(*a*), PPAd). Peaks at 24.2 (CH_2_(*c*)), 27.8 (CH_2_(*e*)), 33.7 (CH_2_(*b*)) and 60.8 ppm (CH_2_(*d*)) are attributed to the PPAd unit, while the peaks at 16.6 (CH_2_(*g*)) and 68.9 ppm (CH_2_(*h*)) are attributed to the PLLA unit. Similarly to the ^1^H-NMR spectra, the disappearance of the peak around 58.9 ppm indicates that PPAd chains react via the OH-terminated end. The absence of peaks between the two carbonyl ^13^C resonance signals further corroborates that block and not random copolymers were obtained.

**Figure 4:**
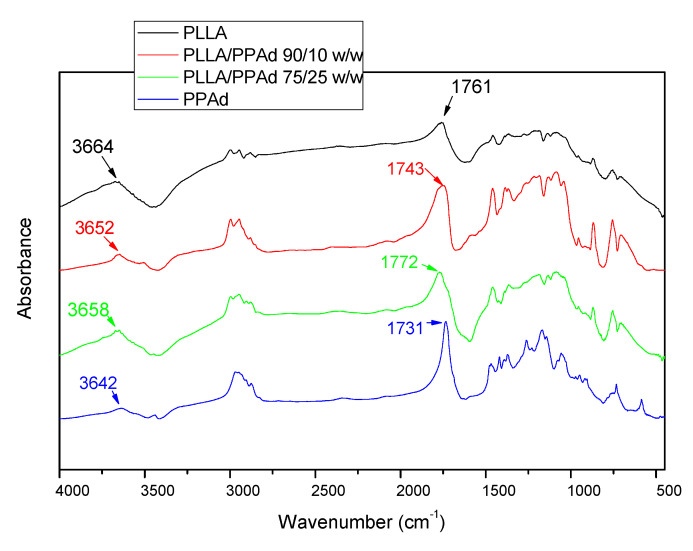
FTIR spectrum of all synthesized polyesters.

The formation of copolymers was also confirmed with FTIR spectroscopy. Figure 4 shows the FTIR spectra of neat PLLA, PPAd and their copolymers. PLLA spectra exhibits a broad absorption peak in the range 3700–3600 cm^−1^ which is assigned to ν(O–H) vibration modes. Hydrogen association can easily occur in hydroxyl groups, resulting in a shift of the corresponding absorption peak to lower wavenumbers and a gradual widening with the increase of the association degree [21]. Furthermore, the two peaks at about 2951 and 3003 cm^−1^ correspond to the ν(C–H) vibration modes of methyl groups. Besides, the δ(C–H) modes of methyl appear around 1458 and 1362 cm^−1^. In the ν(C=O), ν(C–O) and ν(C–C) regions, PLLA exhibits sharp absorption peaks at 1760, 1081, and 1192 cm^−1^ respectively. PPAd exhibits a peak at 3000–2900 cm^−1^ corresponding to the ν(C–H) vibration modes of the methylene groups and sharp absorption peaks at 1731, 1254, and 1192 cm^−1^ corresponding to ν(C=O), ν(C–O) and ν(C–C) respectively. No significant absorption was observed around 3400–3700 cm^-1^, confirming a reasonably high polymerization degree. The main peaks appearing in the homopolymer spectra are retained in the copolymer spectra, without the appearance of any new peaks. These spectra exhibit sharp peaks between 1730 and 1775 cm^−1^ due to vibration of the different ester bonds ν(C=O) and wider peaks at 1200–1100 cm^−1^ attributed to the ν(C–O) bond, indicating that copolymer formation has taken place [22].

**Figure 5:**
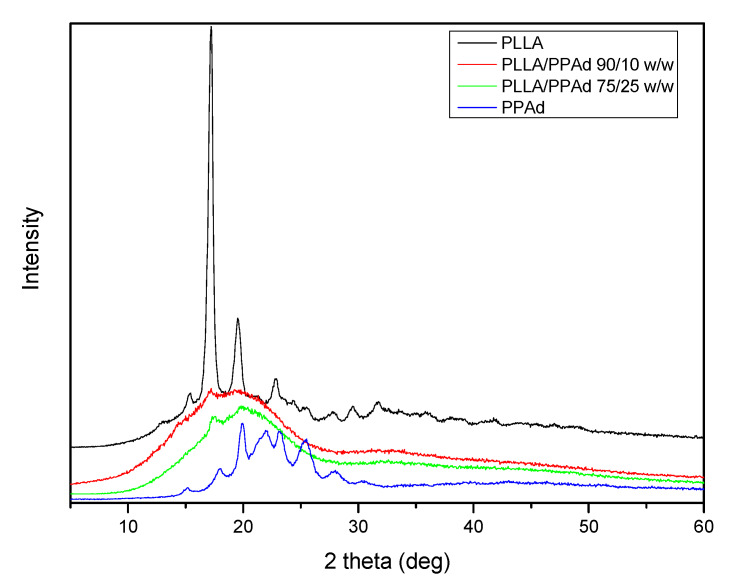
X-ray diffraction patterns of the synthesized polyesters.

The physical state of the prepared copolymers is very crucial for drug release and was studied by XRD and DSC. Figure 5 shows the X-ray diffraction patterns for the synthesized polyesters. Neat PLLA is a polymer with a high degree of crystallinity with characteristic peaks at 2θ = 17.20, 19.60, 22.81 and 31.60°. PPAd shows broad peaks at 2θ = 17.91, 19.90, 22.01, 23.20, 25.40 and 30.50° with low intensity, thus exhibiting a lower degree of crystallinity. Concerning the copolymers, only a small peak at 2θ = 17.20° appears, indicating that the synthesized copolymers have a reduced crystallinity compared to both homopolymers.

The degree of crystallinity of all samples was calculated by wide angle X-ray diffraction patterns using the relative areas under the crystalline peaks (*A*_c_) and the amorphous background (*A*_am_) using Equation (6), according to Lu and Hay [23], and are summarized in Table 1. As expected, PLLA showed the highest degree of crystallinity, with a value equal to 50.6%. In the copolymers the degree of crystallinity decreased with increasing PPAd content. This was expected since PPAd has a low degree of crystallinity (26%) and its incorporation into the macromolecular chains hinders the crystallization of PLLA. Consequently, the copolymer with the highest PPAd amount (25 wt%) has the lowest degree of crystallinity.

(6) $$X_{c}={\left(1-\frac{A_{am}}{A_{c}}\right)}^{-1}$$

**Figure 6:**
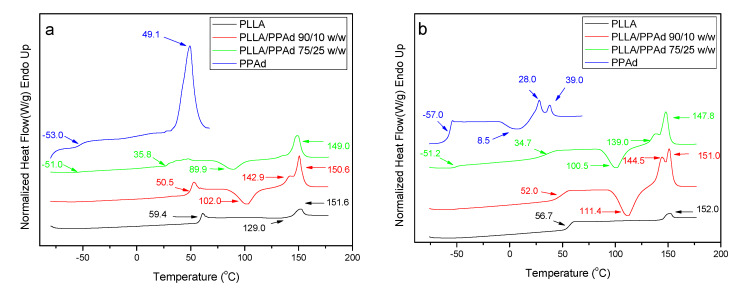
Differential (**A**) first heating and (**B**) second heating scanning calorimetric thermograms of neat polymers and synthesized copolymers.

The thermal properties of all prepared polyesters were also studied. PPAd and PLLA are semicrystalline materials, which showed *T*_g_ values at 59.4 and −53 °C, and *T*_m_ values at 151.6 and 49.1 °C, respectively (Figure 6A). The copolymer with 10 wt% PPAd exhibits also a single *T*_g_ at 50.5 °C, which is very close to that of neat PLLA. On the other hand, copolymer PLLA/PPAd 75/25 *w*/*w* has two glass transition temperatures at −51 °C and 35.8 °C (Table 2). The former corresponds to the PPAd sequences and the latter to the PLLA sequences. This is in good agreement with the literature. Indeed, for a block copolymer consisting of two heterogeneous phases, two glass transition temperatures corresponding to those of the homopolymers are expected [24]. Two *T*_g_ values should also have been recorded for the copolymer with 10 wt% of PPAd. However, since the length of the PLLA segment is expected to be much higher than that of the PPAd segment, the *T*_g_ of PPAd is difficult to be observed. The highly amorphous character of these copolymers, as already suggested from XRD studies, was also confirmed by the appearance of a cold crystallization peak at 102 °C and 89.9 °C for polyesters containing 10 and 25 wt% PPAd, respectively. The recorded *T*_m_ values of PLLA sequence were at 150.6 and 149 °C.

Completely amorphous samples were obtained after melt quenching and their thermograms are presented in Figure 6B. After the second heating process all synthesized copolymers exhibit a double peak at the melting point. The presence of the double peak is due either to the formation of two different crystals formed after the first melting or to the formation of a constituent copolymer where each peak corresponds to a different cluster [16]. The *T*_g_ and *T*_cc_ values are very close to the ones recorded during the first heating with some small differences. As PLLA and PPAd are semicrystalline, the presence of both peaks is expected in the DSC thermographs of the copolymers [25]. However, the *T*_m_ of PPAd was not recorded, due to its low crystallization rate. This is also confirmed by the XRD of PLLA-*b*-PPAd copolymers in which only some peaks of PLLA are recorded (Figure 5).

**Figure 7:**
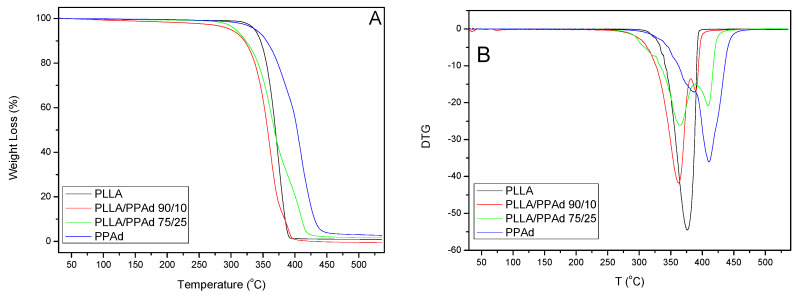
(**A**) Mass loss as a function of temperature with heating rate β = 10 °C/min. (**B**) Differential mass loss as a function of temperature, with heating rate β = 10 °C/min.

Thermal degradation of neat PLLA, PPAd and PLLA-*b*-PPAd 90/10 and 75/25 *w*/*w* copolymers was studied by determining their mass loss during heating, with thermogravimetric analysis (TGA). The mass loss (TG%) and the derivative mass loss (DTG) curves at a heating rate of 10 °C/min are presented in Figure 7. With regard to thermal degradation mechanisms, PPAd is degraded in 2 steps with a maximum decomposition temperature of 410 °C. Aliphatic polyesters generally undergo β- and α- hydrogen bond scissions, yielding low molecular weight volatile products, such as CO_2_, H_2_O, aldehydes, ketones, and allylic products (H_2_C=CH–CH_2_R) [26]. In the case of PLLA, only one degradation step was observed, with a maximum decomposition temperature of 376 °C. The main reaction pathway is an intramolecular transesterification, affording cyclic oligomers [27]. In addition, acrylic acid from cis-elimination as well as carbon oxides and acetaldehyde from fragmentation reactions have also been observed. The onset of thermal degradation of neat PLLA, in spite of exhibiting a faster thermal degradation than the PLLA/PPAd copolymers, is observed at a higher temperature than for copolymers. PLLA/PPAd 90/10 and 75/25 *w*/*w* copolymers follow a two-stage degradation. The lactide-rich sequences degrade first, and the PPAd-rich domains later. However, in neat PLLA the average sequence length of crystallizable lactide units is much larger, leading to a higher and more compact crystalline fraction than in copolymers, hence the onset of thermal degradation is delayed in neat PLLA because of the lower accessibility of lactide units when arranged in close compact crystallite aggregates [28]. Table 3 shows the temperatures at which polyesters exhibited 2%, 50% and 98% mass loss, as well as temperatures with the maximum rate of mass loss.

**Figure 8:**
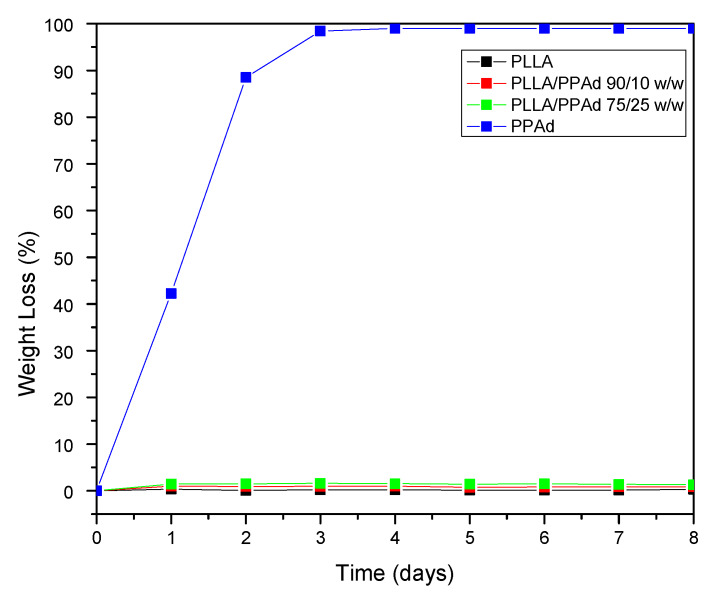
Weight loss versus time of neat PLLA, neat PPAd and PLA/PPAd copolymers at various weight ratios during hydrolysis test in the presence of enzymes at 37 °C and pH 7.

Polyester erosion via the hydrolysis of the ester bonds is one of the main mechanisms of drug release from such polymeric-based systems. Hence, the evaluation of hydrolysis is of crucial importance. As mentioned earlier, the in vivo hydrolysis of PLLA is slow and hence time-consuming [29]. It is thus a common practice to evaluate the hydrolytic degradation in the presence of enzymes [17]. More specifically, in the present work, the enzymatic hydrolysis of PLLA, PPAd and their copolymers was studied using a mixture of *Rhizopus oryzae* and *Pseudomonas cepacia* lipases. These lipases are activated by adsorption on hydrophobic surfaces and consequently are able to cleave ester bonds in the solid phase. As illustrated in Figure 8, neat PLLA showed only limited mass loss during hydrolysis in the presence of enzymes, with approximately 0.3% of the initial mass being lost within the first eight days. No further changes in PLLA mass loss were recorded for up to 18 days (data not shown). On the contrary, PPAd was fully hydrolyzed within three days in the presence of enzymes, indicating that the prepared polyester is fully biodegradable in a short time. The different behavior of polyesters during hydrolysis can be attributed to several factors such as differences in molecular weight, hydrophilic/hydrophobic balance, degree of crystallinity, crystal morphology and melting point [30]. With regard to PLLA/PPAd copolymers, the recorded mass loss rates during enzymatic hydrolysis were near the rates recorded for neat PLLA. Specifically, results in Figure 8 show that as the PPAd content increased in the copolymers, the enzymatic hydrolysis rate slightly increased with approximately 0.9%, and 1.4% of mass loss being recorded within three days for copolymers having 10 and 25 wt% PPAd, respectively. Therefore, it can be safely assumed that the PPAd content of copolymers affected the enzymatic hydrolysis only to a limited extent. The molecular weight as well as the high *T*_m_, which are close to neat PLLA, had probably a more important impact.

**Figure 9:**
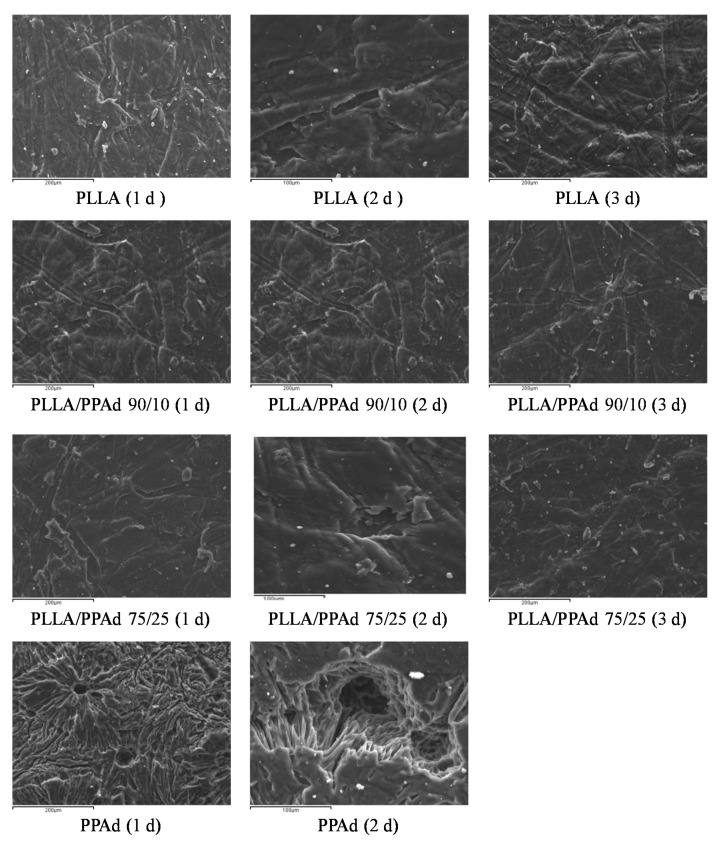
Scanning electron microscopy (SEM) micrographs for surfaces of enzymatic hydrolysis (1–3 days): neat PLLA, neat PPAd, PLLA-*b*-PPAd 90/10 and PLLA-*b*-PPAd 75/25 *w*/*w*.

The morphology of enzymatically hydrolyzed polymers was further examined by SEM. Figure 9 shows the photos taken during the first three days of enzymatic hydrolysis. As shown, the PLLA sample did not change significantly, an expected observation in accordance with the weight loss data. In the case of neat PPAd signs of enzymatic hydrolysis were obvious from the first day: the surface appears to have holes that are progressively growing. Furthermore, the surface became rough with the presence of cracks probably owing to the amorphous fragments of PPAd [30]. With regard to PLLA/PPAd copolymers, structural deterioration on the surface texture of the films was not observed and an appearance close to neat PLLA is clear. The results of SEM micrographs are in full agreement with the mass loss measurements.

### 3.2. Microparticles Characterization

**Figure 10:**
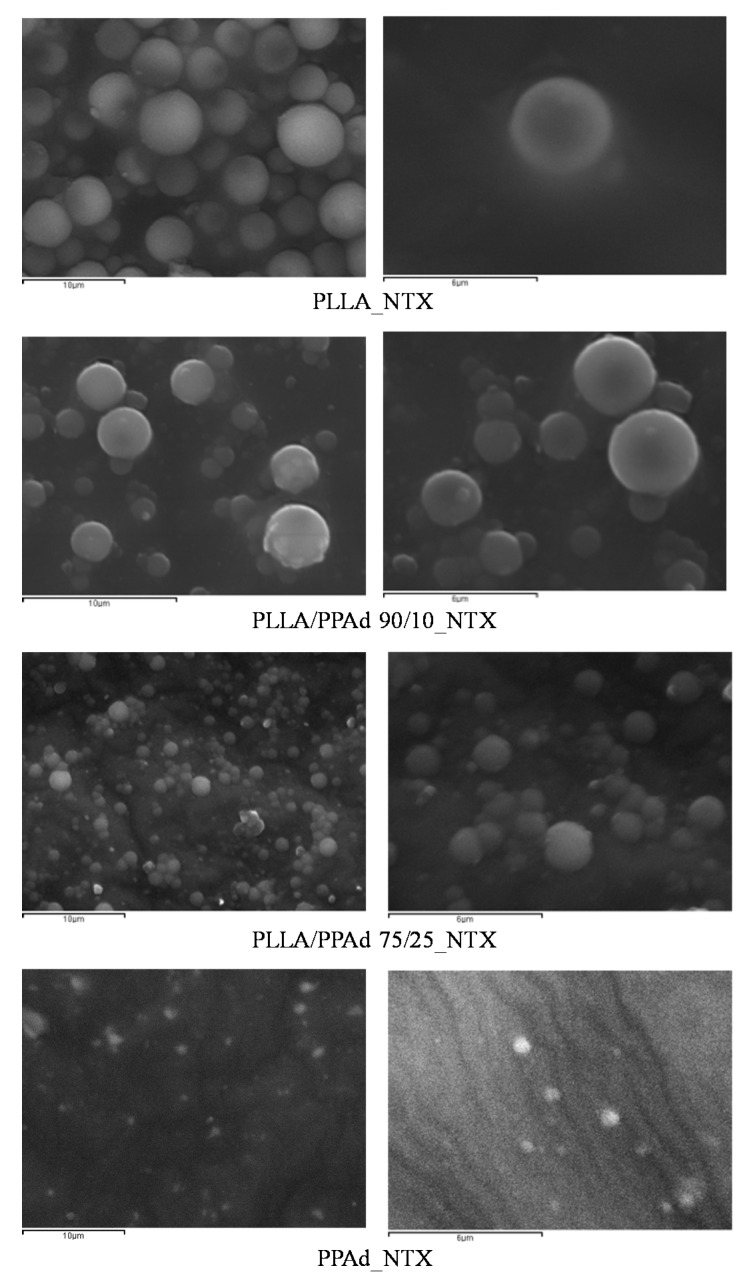
SEM micrographs of prepared microparticles loaded with naltrexone base (NTX).

The prepared copolymers were used for the microencapsulation of NTX drug, applying the single emulsification method. Both the synthesized polyesters and NTX are soluble in DCM. Microparticles were obtained as evidenced by the SEM photos. As observed in Figure 10 all the microparticles had a spherical morphology with smooth surfaces and without agglomeration. Microparticles prepared using PLLA had sizes ranging between 2.6 and 4.5 μm. The sizes for the other microspheres ranged between 2–4 μm for PLLA-*b*-PPAd 90/10, 0.8–2 μm for PLLA-*b*-PPAd 75/25 and 0.4–0.8 μm for PPAd. The size of the microparticles was affected by the PPAd content in the copolymer; i.e., as the content of PPAd increased, the size of the microparticles decreased. Concerning homopolymers this could be attributed to the low molecular weight of PPAd (lower than half the molecular weight of PLLA). Regarding the copolymers, the reduced size could be attributed either to the molecular weight compared with that of PLLA, either to the length of the block segments. Indeed, the length of the PLA segment in copolymer 90/10 is expected to be higher than in copolymer 75/25 and as the length of PLLA segment decreased, smaller particles were formed.

Table 4 presents the yields of microparticles, drug loading, and entrapment efficiency (EE). The yield of microparticles varied from 54.8% (in the case of block copolymer 75/25) to 62.5% (in the case of pure PLLA), indicating high process efficacy. Drug loading varied from 8.75% (in the case of PLLA) to 3.63% (in the case of pure PPAd) and % EE varied from 35.00% in the case of pure PLLA to 14.53% in the case of pure PPAd. Several factors may affect both drug loading and % EE, such as the affinity of the loaded drug with the polyesters, the hydrophobicity of the polymer matrix, the drug solubility in water, drug-drug interactions (i.e., its ability to self-aggregate), etc. It was observed that as the PPAd content increased, the drug loading and EE decreased. This may be attributed to the stereochemical inhibition of NTX and PPAd, as NTX has a relative rigid structure while PPAd is more flexible.

**Figure 11:**
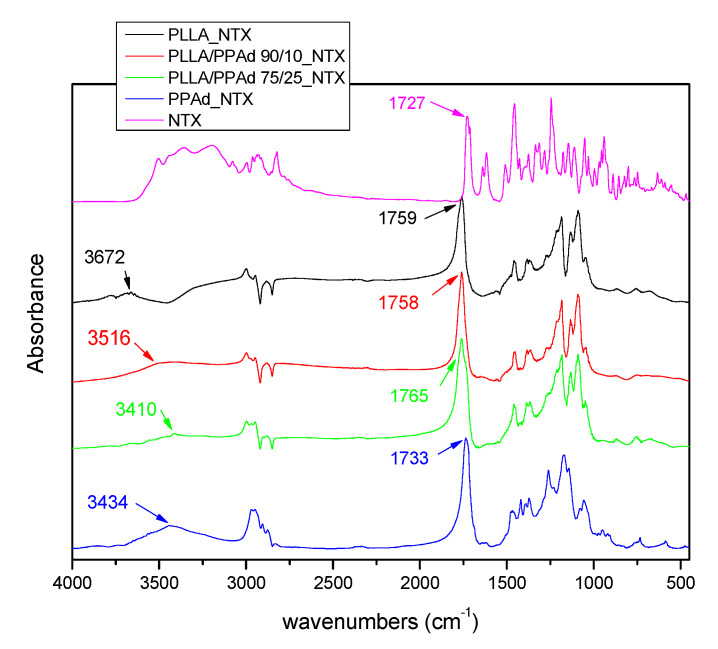
Fourier-transformed infrared (FTIR) spectra of neat naltrexone and prepared naltrexone microspheres with PLLA, PPAd, and PLA-b-PPAd copolymers at several weight ratios.

API solid state properties and physical state have an important role in the chemical and physical stability of the prepared formulations. FTIR was used in order to examine a possible bond formation between the drug and the polyesters in the prepared microparticles. FTIR spectra of NTX-loaded microparticles (Figure 11) showed all the characteristic peaks of NTX and the copolymers. In the FTIR spectrum of NTX, the absorption band at 3000–3100 cm^−1^ is assigned to the ν(C–H) vibrations of the benzene rings, peaks at 1508 and 1617 cm^−1^ to the vibration of ν(C=C) bonds. In the ν(C–C) and ν(C–O) regions, NTX exhibits sharp absorption peaks at 1456 cm^−1^ and 1239 cm^−1^ respectively, a peak at 1727 cm^−1^ corresponding to its ν(C=O) vibration modes, and a broad peak in the range 3200–3500 cm^−1^ due to the existence of OH groups [11]. After the incorporation of NTX, the FTIR spectra of microparticles showed shifts in the region of OH and C=O groups. For example, in PLLA_NTX, a shift was observed from 3664 to 3672 cm^−1^, i.e., to higher wavenumbers, indicating that interactions took place with NTX. Additionally, shifts were also observed in the area of C=O, indicating hydrogen bond formation. Similar shifts were noticeable for all microparticles showing that strong interactions between the drug and the polymeric matrices took place in all cases.

**Figure 12:**
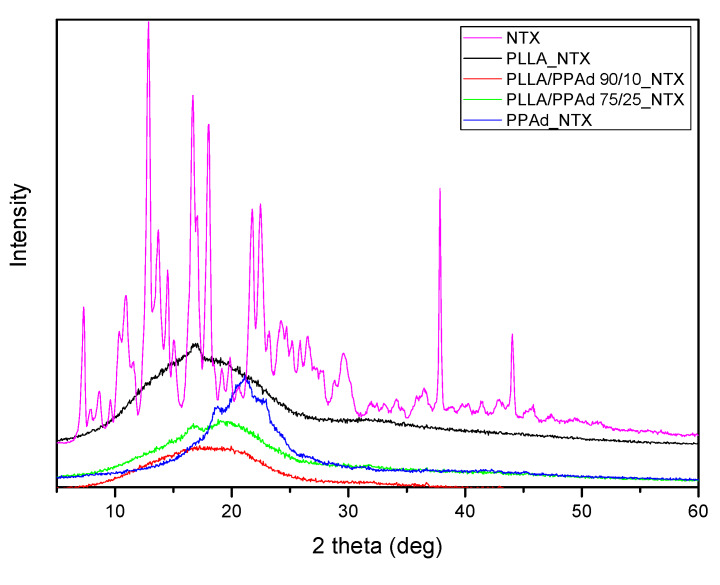
X-ray diffraction (XRD) patterns of neat naltrexone and prepared naltrexone microspheres with PLLA, PPAd, and PLLA-*b*-PPAd 90/10 and 75/25 *w*/*w* copolymers.

XRD was used in order to examine the crystallinity of NTX before and after its encapsulation. As it can be observed in Figure 12, NTX is a highly crystalline drug with two sharp peaks at 2θ = 12.9° and 16.6°, three weaker ones at 21.6°, 22.5° and 37.7° and some much smaller ones [31]. After encapsulation in microparticles no crystallinity of the drug was observed. An exception was the microparticles prepared with PLLA where a small peak at 16.7° was observed. This peak could be attributed either to NTX, meaning that the drug is in a crystalline state in microparticles, either to another type of crystalline formation of the polymer, due to the fact that PLLA has a sharp peak at 17.2°, and the presence of NTX could affect its structure. In microparticles prepared with PPAd it seems that a semicrystalline formulation was obtained mainly ought to the polymer rather than NTX. DSC was further used to confirm these observations.

**Figure 13:**
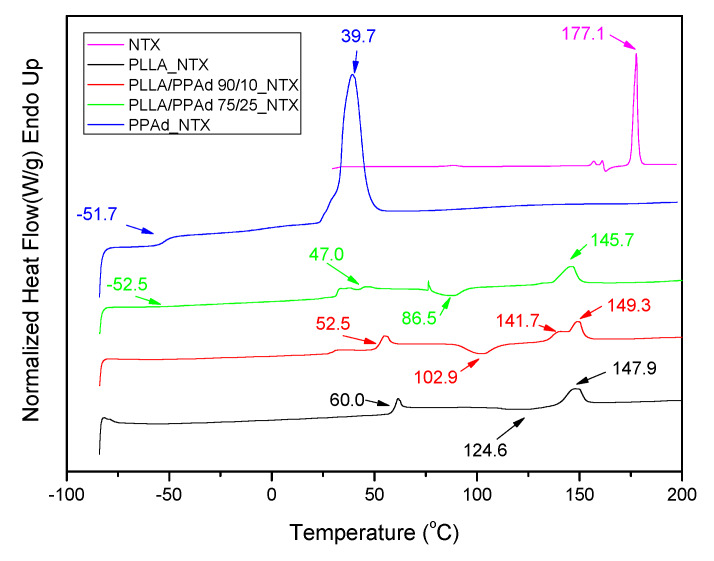
DSC thermograms of neat naltrexone and PLLA, PPAd, and PLA-b-PPAd 90/10 and 75/25 *w*/*w* microspheres loaded with naltrexone.

As shown in Figure 13, a sharp endothermic peak is present for NTX at 177.1 °C [11]. Concerning the DSC curves of the prepared microparticles, the peak of *T*_m_ of NTX was not observed in any formulation indicating that the drug is present in an amorphous state in the microparticles, as was already suggested by XRD. Thermal properties of the polymers after NTX encapsulation, were also studied and some differences compared with neat polymers were observed. In PLLA microparticles, the *T*_cc_ and *T*_m_ values of PLLA changed from 129.0 and 151.6 to 124.6 and 147.9 °C, respectively, after NTX encapsulation. Similar changes in the *T*_g_ and *T*_m_ values of PPAd in microparticles were also observed. *T*_g_ and *T*_m_ of PPAd shifted from −53.0 and 49.1 °C to −51.7 and 39.7 °C, respectively, showing that the NTX also affected PPAd’s structure. For microparticles prepared by copolymers, changes in their characteristic temperatures were also observed; *T*_g_ related to PLLA segment shifted from 50.5 to 52.5 °C, after NTX incorporation in PLA-b-PPAd 90/10 *w*/*w*, and its value appeared at 47.0 °C in PLA-b-PPAd 75/25 *w*/*w*. Concerning the T_m_ value due to the PPAd segment it shifted from 35.8 to 36.1 °C in PLA-b-PPAd 75/25 *w*/*w* and appeared at 30.6 °C in PLA-b-PPAd 90/10 *w*/*w*. From all these it is clear that drug loading affects slightly the thermal properties of the polymers.

**Figure 14:**
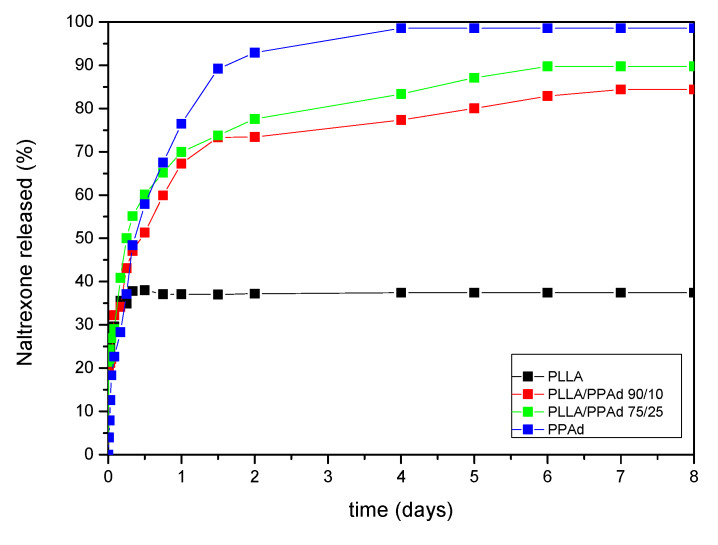
In vitro release profiles of neat naltrexone, along with NTX-loaded microspheres prepared from PLLA, PPAd and PLLA/PPAd copolymers.

Naltrexone’s release profile from the microparticles was studied in phosphate buffered saline, at pH 7.4, containing Tween 20 (final concentration 0.2% *v*/*v*). The dissolution profiles are shown in Figure 14. As it can be observed, there is an initial burst effect in all formulations in the first two hours reaching at about 30%. Thereafter each polymer showed a different behavior. Concerning PLLA, 36% of NTX was released from the microparticles, the release lasted half a day with no further changes after that time. PPAd exhibited a three-stage release. A burst release, as referred previously, with 30% release within the first two hours, followed by a second stage, with a reduced release rate. A total release of 93% was observed within two days. Finally, in the last stage, which lasted until the fourth day, a release of 98.5% was reached. The copolymers showed intermediate release rates, between neat polymers, with PLLA-*b*-PPAd 90/10 and 75/25 release lasting six and five days in which 82% and 90% of the drug was released, respectively. According to these release profiles it is clear that the prepared copolymers have a better profile, compared with PLLA, and could be promising matrices for long-acting release formulations of naltrexone for at least one week.

Release profile is affected by many parameters such as the crystallinity of the drug, the microparticles size, diffusion and thermal properties of polymers. The drug was amorphous in all microparticles, a feature that enhanced its dissolution. The initial burst effect could be attributed to NTX located at the surface of the microparticles, since NTX is hydrophobic and could not be removed during washing steps. After that, diffusion of the drug took place leading to slower release rates [16,17]. Concerning PLLA, Cha and Pitt prepared microparticles of PLLA, PLGA and PCL of Naltrexone base by single emulsification method [32]. It was found that release of NTX from PLLA microparticles was negligible, probably due to steric accessibility of the drug. This result is in agreement with the observation in our study where after the first two hours, no significant release was observed from PLLA microparticles. The same team also used PCL in microparticles preparation and found that NTX was completely released in two days. This was attributed to Fickian diffusion, due to the low *T*_g_ of the polymer [32]. In analogy to PCL, in the present work, NTX release from PPAd microparticles reached 98% in 4 days. This could be due to the low *T*_m_ and *T*_g_ of the microparticles (*T*_g_ was found to be −51.7 °C, a value much lower than 37 °C where the dissolution study was conducted).

The diffusion of NTX through the amorphous polymeric matrix appears to be easier due to the higher mobility of the polyester macromolecules in the amorphous state, allowing an easier penetration of the dissolution medium and, as a result, a faster drug release. Moreover, the thermal properties of the polymer can also affect drug release. When the polymer is exposed to temperatures above the glass transition temperature (*T*_g_), an increase in free volume is observed, which allows for a greater segmental chain mobility and a faster drug delivery. According to the DSC, the *T*_g_ of PPAd is −51.7 °C while PLLA-*b*-PPAd 75/25 *w*/*w* has also a similar *T*_g_, as well as one corresponding to the *T*_g_ of PLLA (Table 2). The copolymer has a lower degree of crystallinity, compared to neat PPAd (Table 1), and thus a higher release rate was expected. However, this is not the case. As it can be seen in Figure 14, PPAd has a higher release rate than PLLA-*b*-PPAd copolymer. This behavior should be attributed to the existence of the PLLA segment in the copolymers, which has a lower mobility than PPAd. This was further confirmed with PLLA-*b*-PPAd 90/10 *w*/*w* copolymer, which, due to the higher PLLA amount, exhibits slower release than the 75/25 *w*/*w* copolymer. The exposure of microparticles with neat PPAd and copolymers with higher PPAd amount at 37 °C, could explain the fast rate of drug release [32,33], indicating that NTX was probably released from microspheres by diffusion. So, it is clear that PLLA, due to its high *T*_g_, delays the release of NTX and its copolymerization with PPAd could enhance the release rate of NTX.

**Figure 15:**
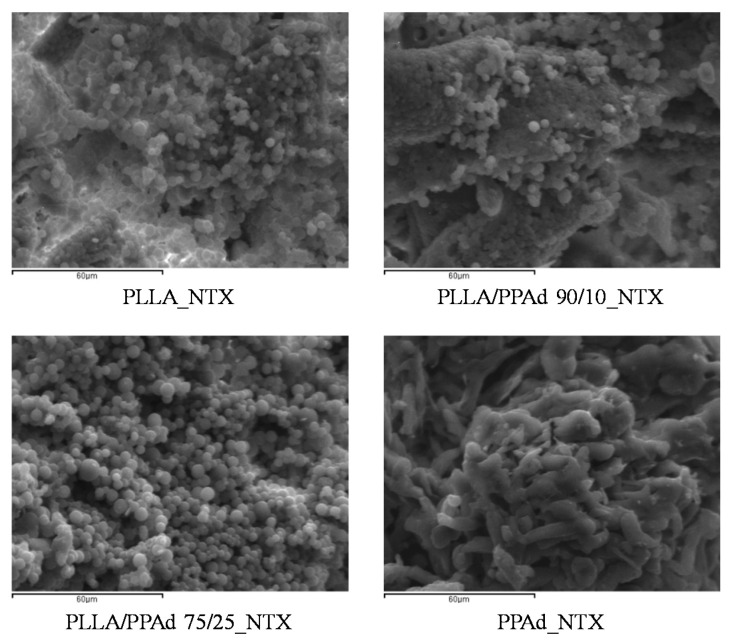
SEM images of NTX-loaded microspheres after 8 days of dissolution.

Figure 15 presents the morphology of the prepared microparticles after 8 days of dissolution, via SEM micrographs. It is clear that the surface of all the microspheres changed since the drug was dissolved. In all cases, aggregation of the microspheres was observed, their surface became rougher, and the normal spherical shape observed in the original microspheres was modified. PPAd microparticles exhibited extensive aggregation and their shape was destroyed. The presence of cavities in the internal morphology of the microspheres has not been confirmed, indicating that there is no erosion of the polymeric matrix. Thus, it is clear that diffusion was the main mechanism of drug release of these microparticles.

### 3.3. Release Data Modeling

According to the previous discussion and based on the experimental evidence, it appears that there is no polymer particle erosion, so the release is dominated by diffusion. This means that the drug release model must be a diffusion-based one. To set up the model the following assumptions are made: (i) the polymer particles are spherical, and they can be assumed to be monodispersed; (ii) the amount of the drug in the bath at the end of the release is much smaller than that corresponding to equilibrium. This is supported by the almost complete release for the case of PPAd. It appears that the non-released drug is trapped in the polymer matrix (maybe in an insoluble crystalline form); (iii) there is no mass transfer resistance to the bulk phase due to the agitation during release. There are approximate expressions for the diffusional release (in the form of power law in time functions) but they refer only to initial release stage [34]. Here the interest is to reproduce the whole release curve so the partial differential equation of transient diffusion must be solved.

There are several levels of complication of the diffusion problem. The first level refers to the mode of diffusion (Fickian/non-Fickian). The second level refers to the possibility of non-uniformity in the polymer structure leading to different release paths with different diffusion coefficients. The third level is the possibility of non-uniform drug concentration along the radius of the polymer particle. Having experimental evidence for the third level and no evidence for the other two, Fickian diffusion with a single diffusion coefficient and non-uniform drug concentration is considered as the starting point to model the data.

The governing equation is:

(7) $$\frac{\partial c}{\partial t}=D\frac{\partial^{2}c}{\partial {r^{\prime }}^{2}}+\frac{2D}{r^{\prime }}\frac{\partial c}{\partial r^{\prime }}$$

where *r*′ is the radial coordinate in the particle, *c* is the local drug concentration in the particle and *D* is the diffusion coefficient in the particle for the mobile fraction of the drug. The particle radius is denoted by *R* and the boundary conditions for Equation (7) are: *c* = 0 at *r*′ = *R*, d*c*/d*r*′ = 0 at *r*′ = 0 and *c*(*r*′) = *c*_o_(*r*′) at *t* = 0. In order to facilitate the mathematical treatment of the problem the following normalization is made: *c* = *C*_ave_*f*(*r*) where *C*_ave_ is the average concentration of mobile drug in the particles, *τ* = *Dt*/*R*^2^ and *r* = *r*′/*R*. The governing equation takes the form:

(8) $$\frac{\partial f}{\partial \tau }=\frac{\partial^{2}f}{\partial r^{2}}+\frac{2}{r}\frac{\partial f}{\partial r}$$

where the boundary conditions are *f* = 0 at *r* = 1, d*f*/d*r* = 0 at *r* = 0 and *f*(r) = *f*_0_(*r*) at *τ* = 0. The function *f*_0_(*r*) denotes the initial drug distribution and by definition fulfills the relation $3\int_{0}^{1}f_{0}\left(r\right)r^{2}dr=1$. According to the above model the release curve is computed as:

(9) $$drug\;released\left(\%\right)=M_{\infty }\left(1-3\int_{0}^{1}f\left(r,t\right)r^{2}dr\right)$$

where *M_∞_* is the final % drug released. The mathematical problem is now completely defined. A semi-analytical solution is possible by transforming the spherical to planar geometry (by introducing the new function *g*(*r*) = *rf*(*r*)) and exploiting the solutions of the planar problem [35]. The final result is:

(10) $$M=M_{\infty }\left(1-\frac{2}{\pi }\overset{\infty }{\underset{i=1}{\sum }}\frac{1}{i}e^{-i^{2}\pi^{2}\tau }\int_{0}^{1}rf_{0}\left(r\right)\sin\left(i\pi r\right)dr\right)$$

It appears that for any profile of the initial drug distribution the functional form of the release curve is:

(11) $$M=M_{\infty }\left(1-\overset{\infty }{\underset{i=1}{\sum }}g_{i}e^{-i^{2}\pi^{2}\tau }\right)$$

In the particular case of uniform initial profile, the coefficients *g_i_* take the form *g_i_* = 6/(*iπ*)^2^.

**Figure 16:**
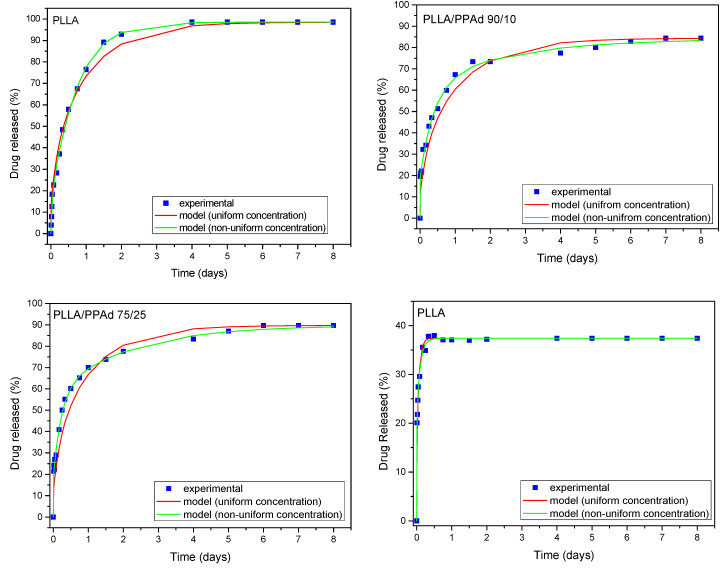
Experimental and model (for uniform and non-uniform drug profile) release curves.

At first the possibility of fitting the experimental release curves assuming a uniform drug distribution will be examined. The fitting variable is the diffusion coefficient *D*. The particle radius *R* for each material is taken from the average diameter data presented in Table 4. The fitting results appear in Figure 16. As it is clear the fitting quality is acceptable only for the case of PLLA. In the other cases there is a systematic deviation between the data and the fitted curves. The next step is to consider non-uniform drug profile. No specific shape of the profile needs to be assumed. The fitting to the data is performed employing relation (11) with fitting parameters the diffusion coefficient *D* and the coefficients *g*_1_, *g*_2_, *g*_3_ (assuming *g_i_* = 0 for *i* > 3). The fitting curves are shown in Figure 16 and a major improvement of the fitting quality (compared to that for uniform profile) is observed. The improvement appears to increase from PLLA to PPAd implying increasing drug profile non-uniformity in the particular order. It is denoted that in all cases the fittings are made using the least square criterion.

The success of the model to describe the data suggests that drug profile non-uniformity is enough to describe the experimental data and the other levels of complication do not have to be invoked. It is worth noticing that the profile of drug that fits the data is not determined directly by this approach. An additional complicated inverse problem must be set to go from the fitting parameters to the actual profile [36]. The values of the diffusion coefficients that fit the data are: 8.67 × 10^−19^ m^2^/s for PPAd, 8.75 × 10^−20^ m^2^/s for PLLA/PPAd 75/25, 1.45 × 10^−20^ m^2^/s for PLLA/PPAd 90/10, 1.55 × 10^−19^ m^2^/s for PLLA. The diffusion coefficients are extremely small; almost ten order of magnitude smaller than those in liquids. It also appears that the diffusivity in neat polymers is higher than in polymer mixtures. The proposed model describes adequately the release data and it is compatible with the experimental observations of particle size and drug distribution non-uniformity.

## 4. Conclusions

PPAd and its copolymers with PLLA were successfully synthesized. According to GPC all polymers exhibited a unimodal distribution and the average molecular weights increased by increasing the percentage of PLLA in the copolymers. XRD measurements showed that PLLA had a higher crystallinity than PPAd, while all copolymers, due to the addition of PPAd segments, exhibited a lower degree of crystallinity than both homopolymers. This also affected their thermal properties. Indeed, DSC study revealed that an increase in PPAd content is associated with a concomitant decrease in the melting point and heat of fusion of copolymers. The enzymatic biodegradation study of polymers showed that PPAd had a very high degradation rate, compared to PLLA. This is probably due to the lower degree of crystallinity of PPAd over PLLA and to its lower melting point. However, due to the low percentage of PPAd in the prepared copolymers with PLLA, their enzymatic hydrolysis is almost similar to that of neat PLLA.

The microparticles prepared from the synthesized polymers for microencapsulation of the Naltrexone drug compound were spherical as illustrated by SEM micrographs, with average diameter ranging between 0.4–4.5 µm, depending on the polymer. From XRD, FTIR and DSC analysis, we concluded that amorphization of naltrexone occurred during microencapsulation, due to the strong interactions taking place between naltrexone and the polymeric matrices. The study of drug release showed that release is probably due to diffusion of NTX from microparticles, since no erosion was detected in nanoparticles by SEM. From dissolution studies it is clear that drug release is directly dependent on the polymer matrix used. Neat PPAd due to its low melting point and *T*_g_ values gave microparticles with the highest release while PLLA the lowest release. Copolymers showed an intermediate release depending on the copolymer composition. Naltrexone release is extended over seven days, indicating that these novel PLLA-*b*-PPAd copolymers could be used in long-acting injectable formulations of naltrexone drug. The fitting of a diffusion-based release model to the experimental release data suggests non-uniform drug loading of the particles.

## Figures and Tables

**Table 1:** Theoretical and calculated compositions, intrinsic viscosities, and molecular weights of the prepared polymers.

Samples	*w*/*w* Ratio	^1^H NMR (mol Ratio)	Exp. *w*/*w* Ratio ^a^	[*η*] (dL/g)	Average *M*_n_ (g/mol)	Average *M*_w_ (g/mol)	*M*_w_/*M*_n_	*X*_c_ (%)
PLLA	100/0	100/0	100/0	0.99	86,000	133,900	1.56	50.6
PLLA/PPAd 90/10	90/10	88/12	85/15	0.77	53,700	79,500	1.48	18.5
PLLA/PPAd 75/25	75/25	74/26	69/31	0.65	47,700	77,400	1.62	13.5
PPAd	0/100	0/100	0/100	0.38	35,000	50,700	1.45	26.0

^a^: experimental ratio calculated from the mol ratios, as calculated from ^1^H-NMR spectra.

**Table 2:** Glass transition temperature (*T*_g_), cold crystallization temperature (*T*_cc_) and melting temperature (*T*_m_) of PLLA, PPAd and their copolymers.

Sample	*T*_g_ (°C)				*T*_c_ (°C)		*T*_m_ (°C)			
	*T*_g1_	*T*_g2_	*T*_g1_′	*T*_g2_′	*T*_cc_	*T*_cc_′	*T*_m__1_	*T*_m__2_	*T*_m__1_′	*T*_m2_′
PLLA	59.4	-	56.7	-	129.0	-	151.6	-	152.0	-
PLLA/PPAd 90/10	50.5	-	52.0	-	102.0	111.4	142.9	150.6	144.5	151.0
PLLA/PPAd 75/25	38.8	−51.0	34.7	−51.2	89.9	100.5	149.0	-	139.0	147.8
PPAd	−53.0	-	−53.0	-	-	8.5	49.1	-	28.0	39.0

Abbreviations: *T*_g_**′** *T*_cc_**′** *T*_m_**′** referring to second heating.

**Table 3:** Data obtained by thermogravimetric analysis of synthesized polymers.

Samples	*T*_d_, 2% (°C)	*T*_d_, 50% (°C)	*T*_d_, 98% (°C)	*T*_max_ (°C)	Residue (%) 500 °C
PLLA	322.4	370.3	390.5	376.3	1.01
PLLA/PPAd 90/10	226.5	357.0	394.5	362.7	-
PLLA/PPAd 75/25	287.0	367.4	455.9	364.3	1.95
PPAd	308.9	404.5	Residue	410.0	2.97

**Table 4:** Characteristics of prepared naltrexone-loaded microparticles such as yield of microparticles (%), drug loading (%), and entrapment efficiency (%).

Sample	Diameter (μm)	Yield of Microparticles (%)	Drug Loading (%)	Entrapment Efficiency (%)
PLLA	2.6–4.5	62.5 ± 2.0	8.75 ± 0.22	35.00 ± 2.02
PLLA/PPAd 90/10	2.0–4.0	61.7 ± 3.6	8.30 ± 0.21	33.21 ± 1.62
PLLA/PPAd 75/25	0.2–2.0	54.8 ± 1.2	5.89 ± 0.22	23.55 ± 1.02
PPAd	0.4–0.8	58.9 ± 4.5	3.63 ± 0.15	14.53 ± 0.82
